# Proposal for Self-Degrading Power Cables Incorporating Graphitic Carbon Nitride to Address Electronic Waste Challenges and Evaluation of Decomposition Efficiencies

**DOI:** 10.3390/molecules30193951

**Published:** 2025-10-01

**Authors:** Satoshi Horikoshi, Kanon Hirota, Nick Serpone

**Affiliations:** 1 Department of Materials & Life Science, Faculty of Science and Technology, Sophia University, 7-1 Kioicho, Chiyodaku, Tokyo 102-8554, Japan; 2 PhotoGreen Laboratory, Dipartimento di Chimica, Università di Pavia, via Taramelli 12, 27100 Pavia, Italy; nick.serpone@unipv.it

**Keywords:** graphitic carbon nitride (g-C_3_N_4_), photocatalyst, electronic waste (e-waste), PVC film, power cable

## Abstract

This study addresses challenges in recycling electronic waste (e-waste) by developing a self-degrading electrical wire coating material using graphitic carbon nitride (g-C_3_N_4_). Two types, melamine-derived carbon nitride (MCN) and urea-derived carbon nitride (UCN), were synthesized and evaluated for their photocatalytic activity by measuring the decolorization rate of rhodamine-B (RhB). UCN demonstrated superior photocatalytic performance compared to the widely used TiO_2_. When incorporated into PVC film, UCN achieved a maximum weight loss of 68% in photodegradation tests after 40 days of irradiation, contributing to reduced environmental impact. A UCN-mixed coating for a vinyl-insulated cable prototype showed that photodecomposition in water facilitated copper wire separation. The study also indicated that water is vital for the decomposition process, while UCN enhanced stiffness and tensile strength of the material without compromising elongation and electrical insulation properties.

## 1. Introduction

The rapid advancement of information and communication technology has led to a continuous increase in the global generation of electronic waste (e-waste). According to the United Nations University, e-waste—defined as discarded electrical and electronic equipment (WEEE)—is projected to reach 62 million tons by 2024. Alarmingly, only 22.3% of this waste is expected to be properly recycled [1]. E-waste encompasses a diverse array of materials, including precious and rare metals, hazardous substances, and persistent polymeric materials. As a result, there is a pressing need for the development of affordable and user-friendly recycling technologies that can effectively recover resources and mitigate environmental impacts [2]. In response to this challenge, the Basel Convention was enacted in 1992 to regulate the international movement of hazardous waste, including e-waste [3]. The convention aims to prevent the uncontrolled transfer of waste from developed nations to developing ones, with a particular focus on e-waste that contains persistent plastics such as PVC and heavy metals.

Among e-waste, electrical wires and cables commonly found in homes and buildings are primarily made of copper, which is highly recyclable, along with a coating primarily composed of polyvinyl chloride (PVC). The annual production of copper-based power cables is estimated to be around 15 to 17.5 million tons [4]. Demand for these cables continues to rise due to the increasing prevalence of electrical appliances [5]. However, the supply of copper is not keeping pace with this demand, making the recycling of copper an urgent concern.

The PVC used in coatings possesses excellent flame retardancy, weather resistance, and insulating properties. However, it is challenging to decompose and can release harmful chlorine-based compounds during thermal breakdown [6]. Additionally, removing PVC from electrical wires necessitates the use of wire strippers, a process that is often time-consuming and labor-intensive. This complexity varies depending on the equipment used and the volume of waste, as wire specifications differ [7]. Therefore, there is a pressing need to develop a recycling method that can selectively recover valuable metals from PVC-containing electrical wires and cables, all while minimizing environmental impact, reducing costs, and facilitating easy recovery. To tackle this challenge, we have previously reported on using microwave technology for the thermal decomposition of insulation materials, which allows for the recovery of copper wires [8]. This method rapidly carbonizes the insulation using microwave heating or discharge, generating minimal by-products such as tar, and significantly lowering CO_2_ emissions. An added benefit is that the wires can be processed without the need for cutting.

It is also essential to develop simple, small-scale recycling methods for e-waste that has been discarded in the natural environment, as well as to facilitate the recycling of e-waste on a smaller scale without relying on specialized equipment. This concept originated from our 1998 study on a self-degrading material created by blending PVC with the photocatalyst TiO_2_ [9]. This material demonstrated self-degradation after 20 days of exposure to sunlight, with the PVC breaking down into Cl^−^ ions and CO_2_. The idea of “plastics with a lifespan” is particularly relevant to the ongoing concerns surrounding microplastics [10] and shows promise for use as a self-degrading material in the environment.

This method, however, presents two significant challenges. Firstly, TiO_2_ is only activated by ultraviolet light with wavelengths below 387 nm, which limits the efficiency of sunlight or indoor light utilization (Issue 1). Secondly, TiO_2_ may persist in the environment after decomposition (Issue 2). To tackle Issue 1, research has focused on developing visible-light-responsive photocatalysts [11], leading to advancements in polymer photocatalysis. For instance, Tang et al. [12] achieved a 40% dechlorination of PVC utilizing uranyl ions (UO_2_^2+^). Moreover, Kynman et al., [13] reported the upcycling of PVC using metal–organic frameworks (CDs/Zr-MOFs), while Ran and coworkers [14] have presented a comprehensive review on the upcycling process.

Many existing studies have primarily concentrated on examining the interaction between photocatalysts and polymers, along with their decomposition behavior, while there has been limited fundamental research on their application to e-waste recycling. Following an earlier study [9], we substituted TiO_2_ with a visible-light-responsive graphitic carbon nitride (g-C_3_N_4_) photocatalyst and investigated its potential for e-waste recycling. This material is a metal-free photocatalyst that poses a low residue risk post-decomposition, which could address Issue 2. Additionally, it can be synthesized from cost-effective raw materials such as melamine and urea [15] and is believed to exhibit high compatibility with PVC [16]. Moreover, g-C_3_N_4_ generates oxidizing species like •OH radicals and O_2_^−^ in aqueous environments, demonstrating a decomposition mechanism akin to that of TiO_2_ [17].

The photocatalytic activity of TiO_2_ photocatalysts is generally limited under visible light [18]; however, advancements have been made to enhance their performance. In this regard, earlier investigations focused on the photocatalytic degradation efficiency of pure PVC [(C_2_H_3_Cl)_n_] and PVC films containing TiO_2_ or modified TiO_2_ as photocatalysts [19]. The degradation efficiency was higher with TiO_2_ addition, mainly due to increased light absorbance, although optical transparency decreased with higher TiO_2_ content. Weight loss was greater in air than in nitrogen, indicating that oxygen was essential for effective photocatalytic degradation. Additionally, studies on a vitamin-C (VC)-modified TiO_2_ photocatalyst [20] showed that VC enhances light absorption and photocatalytic activity. The optimal mass ratio of VC to TiO_2_ for maximum efficiency was 0.5, with the absorption spectrum shifting up to 600 nm due to the formation of a TiIV-VC charge-transfer complex. When exposed to light, the TiIV-VC charge-transfer complex enhances the combined effect of VC and TiO_2_. The excited electrons move from VC to the TiO_2_ conduction band, resulting in the creation of superoxide radical anions. These radicals then interact with nearby polymer chains, speeding up the degradation of PVC through a one-electron reduction of surface oxygens.

Another such development was the modification of TiO_2_ with perchlorinated iron (II) phthalocyanine, known as FePcCl_16_-TiO_2_ [21], which demonstrated a significantly improved absorption capability in the visible light spectrum. Other modifications have also shown promise, including the use of bismuth oxyiodide (BiOI) [22], polyoxometalates (POM) [23], and nano graphite [24]. These variations—referred to as PVC BiOI/TiO_2_, PVC-POM/TiO_2_, and PVC-(Nano-G/TiO_2_), respectively—exhibit enhanced photocatalytic degradation of PVC by broadening the absorption spectra. The effectiveness of these modified photocatalysts largely stems from their ability to enhance the migration and separation of photogenerated electrons within the TiO_2_ matrix. This is achieved through the creation of a built-in electric field at the formed heterojunctions, which helps to inhibit the recombination of photogenerated charge carriers. As a result, these modifications significantly boost the rate of photocatalytic degradation, making them valuable for applications under visible light. In a more recent investigation, Kaur and Kaur [25] synthesized composites of graphene oxide (GO) and nitrogen-doped titanium oxide (N-TiO_2_) in different ratios, one of which (GO:N-TiO_2_ = 1:3) demonstrated a photocatalytic removal efficiency of over 95% degradation of PVC plastic nanoparticles across pH levels 4, 7, and 10 after 3 h of irradiation (tungsten bulb).

This study follows our previous research of 1998 [9], in which we highlighted the photocatalyzed degradation of polymers in aqueous semiconductor suspensions, specifically a TiO_2_-blended polyvinyl chloride film. In the current investigation, we synthesized melamine-derived carbon nitride (MCN) and urea-derived carbon nitride (UCN) and subsequently developed composites by uniformly dispersing these materials within PVC. We evaluated their self-destructive behavior when subjected to light irradiation and created a prototype coating material featuring UCN for a vinyl-sheathed flat-type (VVF) cable (VVF cable, which is widely utilized for indoor electrical wiring in Japan, is characterized by its flat configuration and is constructed from polyvinyl chloride (PVC) insulation and sheathing. The designation "VVF" denotes Vinyl Vinyl Flat, reflecting its composition of two or more conductors, typically made of copper, that are insulated with PVC and subsequently bundled together. This assembly is then encased in a robust outer PVC sheath, providing both protection and functionality in various electrical applications.) designed for residential wiring. Additionally, we explored the recovery of copper wire and the changes in the material’s physical properties. The main goal was to develop a self-destructing electronic material that is both recyclable and practical.

## 2. Results and Discussion

### 2.1. Synthesis and Physical Properties of MCN or UCN Powders

The synthesis of MCN and UCN based on prior studies [26,27,28] proved unproductive as it led to inconsistent reproducibility under our experimental conditions. Consequently, this study commenced with an investigation into the optimal synthesis conditions tailored to our setup. Specifically, we placed 3.00 g of melamine (Fujifilm Wako Pure Chem. Co., Osaka, Japan, 99% special grade, C_3_H_6_N_6_) or 10.00 g of urea (Fujifilm Wako Pure Chem. Co., Osaka, Japan, Guaranteed Reagent, CO(NH_2_)_2_) in a 50 mL alumina crucible with a lid and subjected it to heating in an electric furnace. Preliminary experiments indicated that temperature fluctuations caused by convection within the electric furnace impacted negatively on the synthesis process. To mitigate this issue, we utilized a small electric furnace (Hi Cera Kiln NHK-120 BS-I) to minimize convection effects during the heating process.

**Figure 1:**
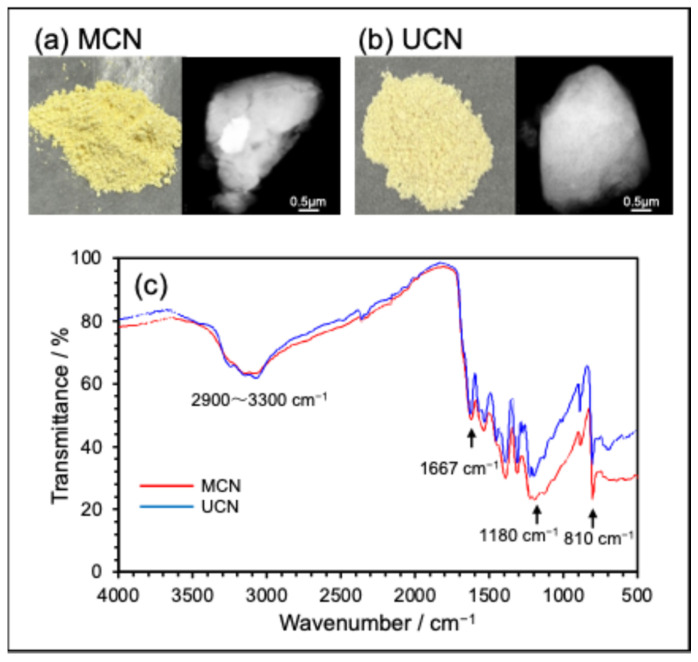
(**a**) The visual characteristics and transmission electron microscopy (TEM) image of MCN powder synthesized under optimal heating conditions; (**b**) The visual characteristics and TEM image of UCN powder synthesized using the same methodology. (**c**) The infrared absorption spectra (FT-IR) of the synthesized MCN and UCN.

Furthermore, we examined the heating profile of the synthesis process. The initial heating rate was set at 19 °C per minute, reaching 190 °C to facilitate the dehydration of the precursor. Subsequently, the rate was decreased to 3 °C per minute until it reached 550 °C, where it was maintained for a duration of 4 h. After calcination, the mixture was allowed to cool naturally at room temperature and atmospheric pressure. This process successfully yielded bright yellow powdered MCN and UCN (see Figure 1a,b). Under these optimal conditions, the synthesis yields were approximately 48% for MCN and around 3% for UCN. The significantly lower yield of UCN is attributable to the melting of urea at approximately 133 °C, which decomposes into ammonia and carbon dioxide upon heating; these gases then evaporate from the system and do not contribute to the reaction.

The chemical structures of the synthesized MCN and UCN were analyzed using FT-IR spectroscopy (Figure 1c). The characteristic absorption peaks of g-C_3_N_4_ include a broad peak in the range of 2900–3300 cm^−1^, attributed to NH stretching vibrations, a peak at 1667 cm^−1^ corresponding to the C=N bond, a broad peak around 1180 cm^−1^ resulting from C–N heterocyclic stretching vibrations, and an in-plane vibration of the triazine ring at 810 cm^−1^ [29]. The isoelectric points (pIs) of MCN and UCN were determined to be 4.7 and 4.8, respectively, both of which are lower than that of Evonik P-25 TiO_2_ (pI = 6.3) [30]. This indicates that g-C_3_N_4_ carries a negative charge in neutral aqueous solutions. Bandgap measurements indicated that MCN has a band gap of 2.8 eV (absorbing light below 443 nm), while UCN has a band gap of 2.7 eV (absorbing light below 459 nm), confirming that both materials exhibit responsiveness to visible light. The particle size (as shown in Figure 1a,b) was larger than that of P-25 TiO_2_ (approximately 30 nm) [30], although the specific surface area was significantly lower. Considering that both particle size and specific surface area are crucial factors influencing photocatalytic activity, the advantages of g-C_3_N_4_ over TiO_2_ are primarily attributed to its superior light absorption properties.

### 2.2. RhB Decolorization Experiment Using Powdered MCN or UCN

**Figure 2:**
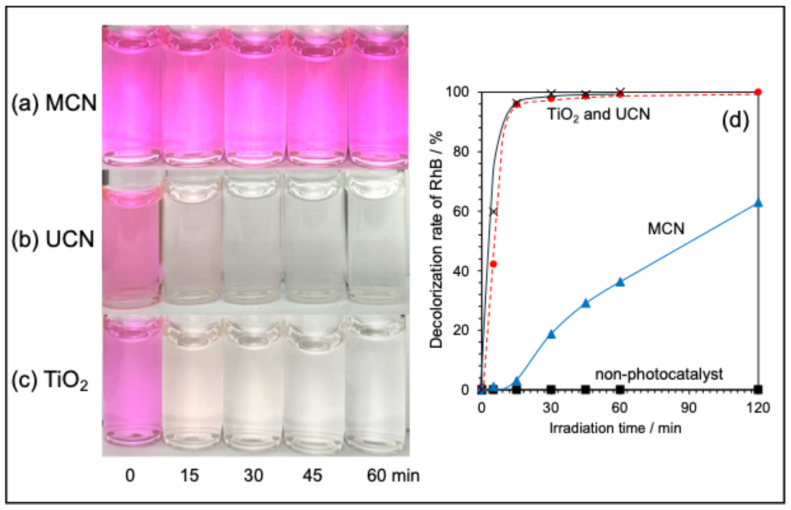
Illustration of the decolorization of RhB aqueous solution through light irradiation using various photocatalysts: (**a**) MCN, (**b**) UCN, and (**c**) P-25 TiO_2_. Panel (**d**) presents a comparison of the decolorization rate (DC%), determined from the reduction rate of the absorption peak at 552 nm in the RhB aqueous solution.

The photocatalytic activity of the synthesized MCN and UCN was assessed in comparison to the well-known photocatalyst P-25 TiO_2_, using the decolorization rate of a rhodamine-B (RhB) aqueous solution as a measure. Figure 2 illustrates the changes in color and the decolorization rate of the RhB solution over time.

In the case of MCN, slight decolorization was visually noted after 60 min of light irradiation (Figure 2a), and the absorbance at 552 nm decreased by approximately 36% from the initial value (Figure 2d). With continued light exposure, the solution became completely transparent after 4 h, leading to the disappearance of the absorption peak at 552 nm. Conversely, for UCN, the solution achieved transparency after just 30 min of light irradiation (Figure 2b), with a 96% decrease in absorbance observed after 15 min; the peak vanished entirely after 30 min as shown in Figure 2d. Notably, this outcome closely mirrored that of P-25 TiO_2_, which exhibited a 96% decrease in absorbance after the same 15-min period, resulting in the solution turning completely transparent after 30 min as depicted in Figure 2c). These findings indicate that the photocatalytic activity order for RhB is TiO_2_ ≈ UCN ≫ MCN. The pseudo-first-order reaction rate constants for up to 60 min of light irradiation were *k* ≈ 0.15 min^−1^ for TiO_2_, *k* ≈ 0.12 min^−1^ for UCN, and *k* ≈ 0.008 min^−1^ for MCN.

The isoelectric point (pI) of P-25 TiO_2_ is approximately 6.3, positioning it close to the pH of the RhB aqueous solution (around 6.5). Hence, the surface of P-25 TiO_2_ is electrically neutral, resulting in minimal electrostatic repulsion with the cationic dye RhB. Since photocatalytic reactions primarily occur via oxidative species generated near the catalyst surface, the ability for the target substance to adsorb onto the catalyst surface plays a critical role in the efficiency of decomposition [31]. In contrast, UCN has an isoelectric point of pI = 4.8, rendering its surface negatively charged in a pH 6.5 aqueous solution. Consequently, cationic RhB molecules are readily electrostatically adsorbed onto the UCN surface. This variation in adsorption is believed to account for the rapid decomposition of RhB by UCN, despite its less favorable specific surface area.

On the other hand, we considered the reason for the UCN ≫ MCN relationship. UCN has an ultrathin nanosheet structure, giving it a much larger surface area than bulk or mesoporous carbon nitride MCN [32]. Furthermore, the thin structure of UCN is known to speed up the separation and transport of charge carriers and reduce the recombination of photogenerated electrons and holes [32]. This improves the quantum efficiency of the photocatalytic process [32]. Additionally, UCN exhibits more uniform dispersion in aqueous solutions than MCN, which is thought to improve its contact with RhB molecules.

### 2.3. Preparation of MCN- or UCN-Blended PVC Films and Assessment of Their Degradation

**Figure 3:**
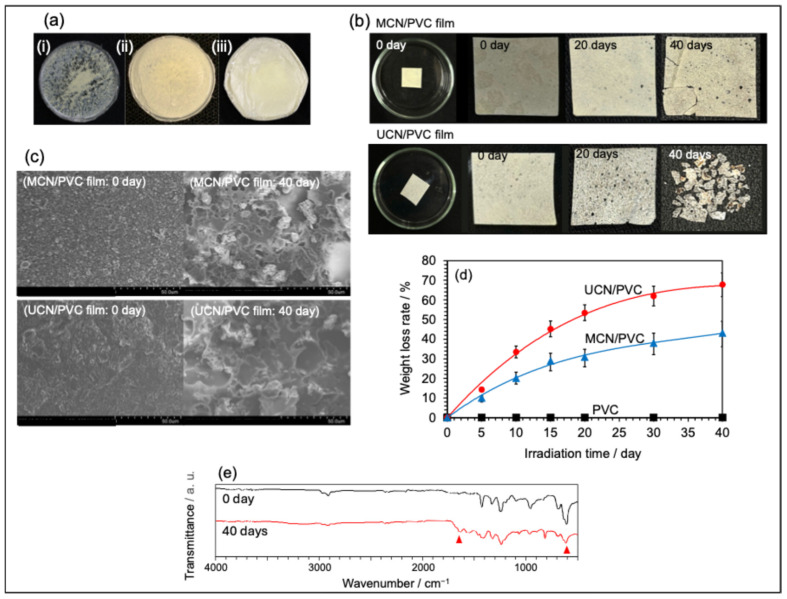
(**a**(**i**)) illustrates the appearance of PVC film with non-uniformly dispersed UCN. (**a**(**ii**)) shows the appearance of PVC film containing uniformly dispersed MCN (MCN/PVC film). (**a**(**iii**)) depicts PVC film with uniformly dispersed UCN (MCN/PVC film); Panel (**b**) presents a comparison of morphological changes due to light irradiation over 0, 20, and 40 days for both MCN/PVC film and UCN/PVC film (10 × 10 mm); Panel (**c**) features SEM images of the surfaces of MCN/PVC film and UCN/PVC film at 0- and 40-days post-irradiation; Panel (**d**) illustrates the changes in weight loss rate as a function of light irradiation time for MCN/PVC film, UCN/PVC film, and PVC film without photocatalyst (PVC); Lastly panel (**e**) details the changes in the infrared absorption spectrum (FT-IR) related to the photodegradation of UCN/PVC film (Red triangle points to the 610 cm^−1^ and 1670 cm^−1^ peaks).

Next, we evaluated the photodegradation behavior of PVC films containing MCN or UCN (MCN/PVC film or UCN/PVC film). PVC films were prepared according to the following procedure, based on previous research [9]. Solid particulates of polyvinyl chloride (PVC; -(CH_2_CHCl)*_n_*-; Shin-Etsu Chemical Co., Ltd., Tokyo, Japan, molecular weight ca. 33,000) were used as raw material for PVC film. PVC powder (100 mg) was placed in a glass dish (φ = 30 mm) containing tetrahydrofuran (THF; 25 mL) and completely dissolved by sonication. Next, 5 mg of MCN or UCN powder was added in the dark and dispersed again by sonication. The THF was then allowed to evaporate naturally in the air at room temperature for 12 h, yielding PVC films containing MCN or UCN. However, this method resulted in aggregation of MCN or UCN in the film, resulting in unevenly dispersed films (Figure 3a(i)). The reason for this was thought to be that the evaporation rate of THF was very fast, which promoted re-aggregation of particles. Therefore, we attempted to improve the dispersibility by controlling the evaporation rate.

In the improved method, 5 mg of MCN or UCN was added to a solution comprising 100 mg of PVC and 25 mL of THF. This mixture was then dispersed for 10 min using a Mitsui Electric Seiki ultrasonic homogenizer (UX-300). Afterward, the dish was covered with aluminum foil containing small holes, allowing for the gradual evaporation of THF in a refrigerator set at 5 °C. This technique successfully produced PVC films with 5 wt.% MCN or UCN homogeneously dispersed throughout (see Figure 3a(ii),a(iii)). These films will be referred to as MCN/PVC film and UCN/PVC film, respectively.

To assess the decomposition behavior of the prepared films, a 10 mm square piece of either MCN/PVC or UCN/PVC film was immersed in a dish containing 7.0 mL of ion-exchanged water and subjected to irradiation with an LED black light (wavelength, 375 nm). Water level marks were marked on the side of the Petri dishes to monitor changes. To minimize evaporation during prolonged exposure, the water level was checked daily, and ion-exchanged water was replenished, as necessary. All experiments were conducted under static conditions, and each condition was repeated three times. The average values obtained were used for analysis.

Figure 3b illustrates the changes in film morphology and weight over exposure time. The MCN/PVC film experienced a gradual decrease in thickness with increasing exposure, accompanied by signs of decomposition, including the emergence of holes on the surface. Similarly, the UCN/PVC film displayed comparable degradation behavior, particularly after 40 days of exposure, with the film fragmenting into smaller pieces.

To further assess the degradation uniformity of the MCN/PVC and UCN/PVC films, their surfaces were examined using a scanning electron microscope (SEM), as shown in Figure 3c. Prior to exposure, both films exhibited a uniform PVC surface, irrespective of the type of MCN or UCN present. After 40 days of exposure, holes began to appear on the film surface, indicating progression in the decomposition process, along with visible peeling. In the case of the UCN/PVC film, degradation initiated around the UCN particles, subsequently leading to the expansion of holes, formation of cracks, and the eventual disintegration of the entire film. Notably, the quantity of UCN particles on the film surface decreased significantly after 40 days, suggesting that UCN itself may have also undergone decomposition during the degradation process. The ion-exchanged water remained transparent post-degradation, with no UCN particles discernible, indicating that during the PVC degradation process, oxidative species such as •OH radicals generated by UCN photoexcitation contributed to the breakdown of both PVC and UCN.

In our previous study, we found that photodecomposition of PVC film containing TiO_2_ resulted in significant PVC degradation due to the strong oxidizing properties of TiO_2_. However, the ion-exchanged water became cloudy following decomposition, indicating that TiO_2_ nanoparticles persisted in the water. The small particle size of TiO_2_ complicates filtration, increasing the risk of its lingering in the environment. While stability is typically a desired trait in photocatalysts, the observation that UCN decomposes under prolonged irradiation in this study could be beneficial for minimizing environmental impact. The use of UCN demonstrates a potential solution to the concern of photocatalysts remaining in the environment.

Figure 3d illustrates the weight loss of PVC films over time. The MCN/PVC film demonstrated a weight loss of 20% after 10 days, 31% after 20 days, and 43% after 40 days. In contrast, the UCN/PVC film showed a weight loss of 34% after 10 days, 53% after 20 days, and reached a maximum of 68% after 40 days. When a pure PVC film was subjected to the same light irradiation, the weight change recorded was less than 1%, indicating that light irradiation alone did not significantly contribute to PVC decomposition. This finding aligns with the RhB decolorization experiment, suggesting that UCN exhibits superior photocatalytic activity for the decomposition of PVC compared to MCN.

The elemental composition of the film surface analyzed through SEM-EDX is presented in Table 1. Notably, oxygen was undetected on the surfaces of the PVC films containing MCN or UCN prior to light irradiation; however, its presence was confirmed following the irradiation process. This phenomenon is believed to result from the oxidation of PVC by active oxidizing species, such as •OH radicals, generated from MCN or UCN during light exposure, which introduces oxygen to the surface.

To determine whether dechlorination occurred during the photodegradation of PVC film, the concentration of chloride ions in the water within the Petri dishes was assessed using HPLC after 40 days of exposure. The UCN/PVC film exhibited a chloride ion concentration of 7.8 mM, whereas the MCN/PVC film showed a significantly higher level of 24.2 mM. This indicates that the photodegradation of both MCN and UCN-blended PVC films involved oxidative degradation of the PVC hydrocarbon chains along with dechlorination, resulting in structural collapse of the film. Additionally, FT-IR analysis of the UCN/PVC film after 40 days of exposure (Figure 3e) revealed a noticeable decrease in the peak at 610 cm^−1^, which is associated with C–Cl stretching vibrations, and the emergence of a new peak at 1670 cm^−1^, corresponding to the amide-derived C=O bond in –CO–NH–. These findings suggest that UCN itself undergoes oxidation while facilitating the dechlorination of PVC.

### 2.4. Proposed Decomposition Mechanism of UCN-Blended PVC Film

Based on these results, we propose the following decomposition mechanism for PVC film. UCN is photoexcited by irradiation with light l < 459 nm, generating holes (h^+^_VB_) and electrons (e^−^) (chemical reaction 1). The generated h^+^_VB_ generates •OH radicals from water molecules (chemical reaction 2), and e^−^ generates oxidatively active species such as the superoxide anion (O_2_^−^) from oxygen molecules (chemical reaction 3). These active species promote PVC decomposition. The C–Cl bond (328.4 kJ mol^−1^) has the lowest bond energy in the PVC molecular chain, and is preferentially cleaved by •OH radicals, leading to dechlorination (chemical reaction 4a). Direct decomposition of PVC by h^+^_VB_ may also occur (chemical reaction 4b), but its decomposition effect is thought to be less significant than that of •OH radicals [9]. Although photocleavage of C–Cl bonds require ultraviolet light of 365 nm or less, the maximum wavelength of the LED black light used in this experiment was 375 nm, making direct photocleavage of the C–Cl bond unlikely. Therefore, decomposition is thought to be primarily dependent on the photocatalytic reaction of UCN. Chlorine atoms cleaved by dechlorination are released into water as Cl^−^ ions, and partial dechlorination of the PVC structure promotes chain decomposition of the molecular chain. Furthermore, scission of carbon chains by •OH radicals also progressed, leading to various alcohol and aldehyde intermediates, ultimately resulting in oxidative decomposition to carbon dioxide (chemical reaction 5). 
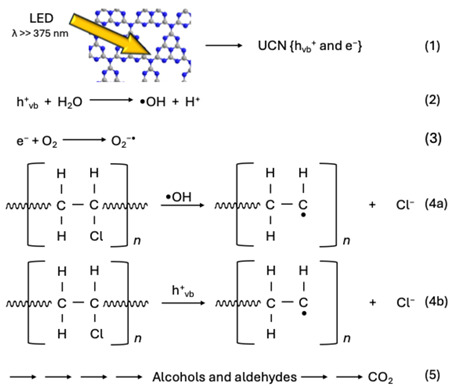


### 2.5. Material Strength of UCN/PVC Film

The results indicate that incorporating UCN is the most effective approach for decomposing a PVC film. Therefore, we assessed the impact of UCN addition on the material strength of PVC film. A comparison was made between PVC film without UCN and PVC film containing 5 wt.% UCN. Undecomposed samples were cut into pieces measuring 10.0 × 15.0 mm and subjected to material testing (as shown in Table 2). We measured the film thickness at five random points, yielding average values of 0.04 mm for the UCN-blended PVC film and 0.02 mm for the unblended PVC film. The maximum stress and elastic modulus of the UCN-blended PVC film were both superior to those of the unblended variant, confirming an increase in material stiffness. This significant enhancement in elastic modulus is attributed to the reduction of intermolecular interactions caused by the incorporation of UCN particles into the PVC matrix, along with the stress-dispersing effect of the UCN’s layered structure [20]. Conversely, while the elongation decreased with increased rigidity, the material still maintained adequate mechanical properties for practical applications as a PVC product. Thus, the enhancement in material strength from adding UCN contributes to achieving both degradability and practicality.

### 2.6. Fabrication and Disassembly Evaluation of MCN or UCN Mixed Wire and Cable

We developed a new material by blending UCN with the PVC sheath of a VVF cable and assessed its decomposition efficiency as a model for e-waste. Our investigation focused on methods to integrate UCN into the PVC sheath, following the fabrication technique for UCN/PVC film. Initially, we employed THF as the solvent; however, since the material was not pure PVC and contained plasticizers, we encountered challenges with volatilization due to persistent high viscosity, and vacuum drying did not lead to complete removal. Consequently, we transitioned to using dimethyl formamide (DMF) as the solvent. The PVC sheath was softened at 85 °C, dissolved in DMF, and allowed to stand at 25 °C and atmospheric pressure for 24 h, which resulted in complete volatilization and hardening. The slower volatilization rate of DMF compared to THF prevented UCN aggregation, even during drying at room temperature.

**Figure 4:**
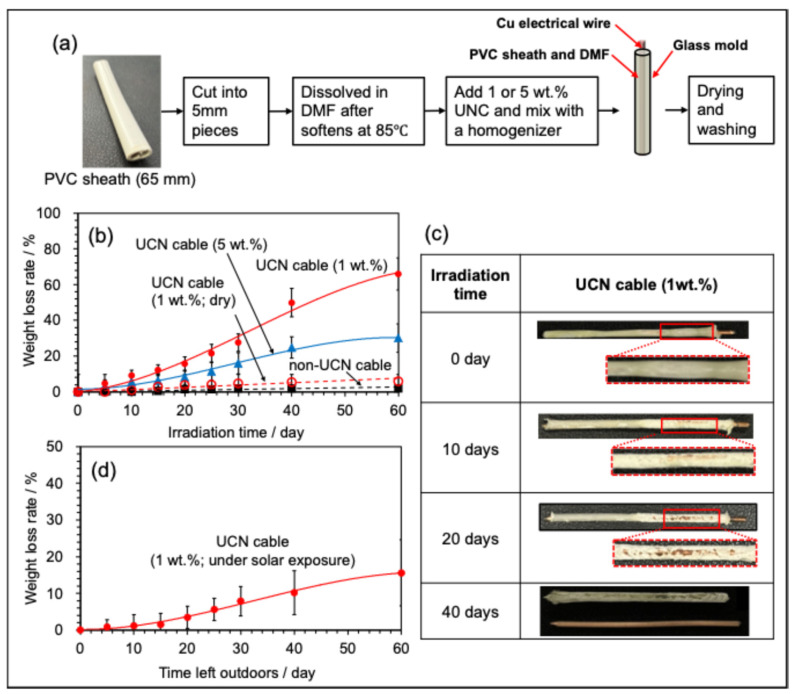
Photographs illustrating the changes in weight and shape during the decomposition of UCN cables in water when exposed to black light; (**a**) flow diagram of creating a UCN-blended simulated power cable; (**b**) The graph depicts the weight loss rate in relation to light exposure time for UCN cables with 1 wt.% and 5 wt.% UCN, as well as for a copper cable that does not contain UCN (non-UCN cable), along with the weight loss rate under anhydrous conditions; (**c**) The images show the changes in shape over time for UCN cables containing 1 wt.% UCN, with light exposure durations of 0, 10, 20, and 40 days; (**d**) The graph illustrates the weight loss rate as a function of sunlight exposure time for UCN cables containing 1 wt.% UCN.

After optimizing the sheath material, we proceeded to fabricate a UCN-blended simulated power cable (referred to as the UCN cable). A 65 mm segment of the PVC sheath from a VVF cable was cut into approximately 5 mm pieces, placed inside a glass container, and heated to 85 °C to soften it. This material was then dissolved in 10 mL of DMF, and UCN was incorporated at concentrations of 1 wt.% or 5 wt.%. The mixture was thoroughly dispersed using a homogenizer. Subsequently, a 65 mm copper wire extracted from a VVF cable was positioned centrally within a glass mold measuring 3 mm in inner diameter and 60 mm in length. The UCN-infused PVC sheath solution was then poured around the wire and allowed to stand at 25 °C for 24 h under atmospheric pressure. Afterward, the cable was washed with methanol and ion-exchanged water and dried under reduced pressure for 12 h to yield a power cable featuring a UCN-containing sheath, known as the UCN cable (see Figure 4a below for a flow diagram of creating a UCN-blended simulated power cable).

For the decomposition experiment, a UCN cable was placed in a deep-bottom Petri dish containing 100 mL of ion-exchanged water and subjected to irradiation from above using an LED black light (see Section 3.2). To determine the weight change, the weight of the copper wire was measured beforehand, and this value was subtracted from the total weight after light irradiation to compute the weight loss rate of the sheath. For comparison, a VVF cable without UCN was also evaluated under the same conditions.

The cable containing 5 wt.% UCN exhibited a weight loss of 5.6% after 10 days, 8.1% after 20 days, 25% after 40 days, and 30% after 60 days (Figure 4b). Conversely, the cable with 1 wt.% UCN showed a weight loss of 9.2% after 10 days, 15.6% after 20 days, 50.0% after 40 days, and 66% after 60 days. Surprisingly, the lower concentration resulted in a higher degradation rate (Figure 4b). This phenomenon may be attributed to aggregation or uneven dispersion caused by the higher UCN concentration, which hampers degradation efficiency. However, a definitive hypothesis has not yet been established. If uniform dispersion can be achieved in the future using a kneader or other methods, it may be possible to achieve significant degradation even at a 5 wt.% concentration. Additionally, the weight loss rate of the VVF cable without UCN after 60 days was merely 2.0%, indicating that UCN is a critical factor influencing the degradation rate.

To assess the impact of water on the decomposition of UCN cables, we conducted a photodecomposition experiment using a 1 wt.% UCN cable placed in a water-free Petri dish (Figure 4b). Even after 60 days of irradiation, the weight loss was a mere 5.8%, which is one-eleventh of the decomposition rate observed in ion-exchanged water (66%). This reduced rate is likely due to the suppression of oxidatively active species, such as •OH radicals, in dry conditions, which significantly lowers the efficiency of decomposition.

A 1 wt.% UCN cable was immersed in ion-exchanged water (7 mL) and placed under sunlight on the rooftop of Building 4 at Sophia University in Tokyo, irrespective of the weather conditions. The experiment was conducted from 25 October to 23 December 2024, totaling approximately 356.6 h of exposure, which included 18 sunny days, 29 cloudy days, and 13 rainy days (Figure 4d). The Petri dishes were positioned under awnings to prevent rainwater intrusion while being strategically angled to maximize sunlight exposure. In comparison to the exposure time under LED black light (1440 h), the outdoor exposure resulted in about one-quarter of the decomposition rate (15.6% at 60 days). Nevertheless, the weight loss rate indicated that decomposition proceeded effectively even in natural environments, due to the influence of sunlight, including ultraviolet light (see Figure 4c).

### 2.7. Material Strength of UCN Cable

The impact of UCN addition on the material properties of UCN cables was assessed with respect to maximum stress, modulus, and elongation. The analysis utilized a UCN-containing PVC sheath film (UCN/PVC-sheath film) prepared by dissolving PVC sheath in DMF, dispersing UCN, and subsequently pouring the resulting solution into a Petri dish with a 3.0 mm inner diameter. For comparative purposes, a UCN-free film was also produced. The thickness of the films was measured at five random locations, yielding average values of 0.65 mm for the 1 wt.% UCN/PVC-sheath film, 0.68 mm for the 5 wt.% UCN/PVC-sheath film, and 0.49 mm for the UCN-free film (see Table 3).

No significant alterations were noted in maximum stress and modulus with the addition of UCN, indicating that the fundamental strength of the PVC sheath remained intact. However, a decrease in elongation was observed alongside UCN incorporation. Specifically, the tensile strength for the 1 wt.% UCN/PVC sheath film was determined to be 595.6 MPa, while the 5 wt.% UCN/PVC sheath film demonstrated a tensile strength of 356.2 MPa, both lower than that of the non-UCN film. This reduction in elongation is attributed to the UCN layer structure infiltrating the PVC molecular chains, which affects the crystalline structure and molecular orientation, thereby constraining molecular chain movement and enhancing stiffness. Nevertheless, the elongation of the UCN-containing film meets the requirement of 120% or higher elongation for PVC sheaths in indoor electrical wiring cables (600 V vinyl-insulated vinyl-sheathed cables), as stipulated in the Japanese Industrial Standard JIS C 3342 [33], thus ensuring its suitability for practical applications. Additionally, electrical conductivity measurements for the 1 wt.% and 5 wt.% UCN-mixed coatings indicated zero conductivity, confirming that electrical insulation was effectively maintained.

## 3. Materials and Methods

### 3.1. Synthesis of MCN or UCN

The synthesis methods for MCN and UCN are thoroughly detailed in Section 2.1. To assess post-synthesis properties, the zeta potential was evaluated using dynamic light scattering (DLS; Otsuka Electronics DLS-8000 series, Tokyo, Japan), and the isoelectric point was subsequently calculated. The pH was adjusted with 1.0 M aqueous acetic acid and 0.1 M aqueous sodium hydroxide. Additionally, the bandgap was measured diffuse reflection measurements using a JASCO Co. UV/Vis absorption spectrophotometer, Tokyo, Japan (V-760) equipped with an Integrating Sphere Unit ISV-922. Bandgap values were determined using the provided software. Particle size was examined using a high-resolution scanning analytical electron microscope (JEOL JEM-ARM200F Thermal FE STEM, Tokyo, Japan).

### 3.2. Decolorization Experiment of RhB Using Powdered MCN or UCN

**Figure 5:**
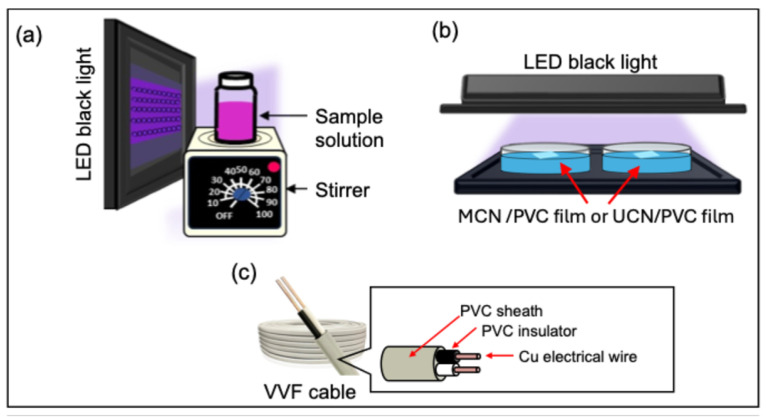
(**a**) Illustrative diagram of the photocatalytic decolorization test utilizing an aqueous solution of rhodamine-B (RhB); (**b**) Illustrative diagram depicting the underwater photodegradation experiment involving PVC film integrated with MCN or UCN (MCN/PVC film or UCN/PVC film); (**c**) Illustrative diagram of the structure of a VVF cable, highlighting the arrangement of the PVC sheath and copper wire.

The photocatalytic activity of MCN and UCN was assessed by measuring the decolorization rate of an aqueous solution of rhodamine B (RhB). For comparison purposes, P-25 TiO_2_ (Evonik), a widely used photocatalyst, was also utilized. In this experiment, 50 mg of each photocatalyst was added to 50 mL of a 0.01 mM RhB aqueous solution (pH = 6.5) within a 100 mL Pyrex screw cap vial. Initially, the sample was dispersed using ultrasonication for one minute, followed by magnetic stirring for 30 min. Subsequently, the sample was irradiated with a Sankyo Electric LED black light (27 W, FPL27BLB, Kanagawa, Japan) (see Figure 5a).

The spectral characteristics of the light source were assessed using an Ocean Optics MAYA2000PRO spectrometer, Tokyo, Japan, which confirmed a distinct single spectrum with a peak wavelength of 370 nm. Throughout all experiments, the distance between the sample and the light source remained constant, with the light intensity maintained at 6.0 mW cm^−2^.

Following irradiation, the photocatalyst was separated from the suspension through centrifugation and subsequently filtered using a 0.2 μm syringe filter. The absorbance of the resulting RhB aqueous solution was assessed with a JASCO Co. UV/Vis absorption spectrophotometer model V-760, Tokyo, Japan. The decolorization rate (*DC*%) was then calculated based on the change in absorbance at the maximum wavelength of 552 nm in the visible region, according to (Equation (6)):

(6) $$DC\%=\left(\frac{C_{0}-C_{t}}{C_{0}}\right)\times 100$$

where *C*_0_ is the initial absorbance, and *C_t_* is the absorbance after light irradiation.

In a preliminary experiment, an aqueous solution of RhB containing a photocatalyst was stirred in the dark for 60 min, showing no change in absorbance at 552 nm. Additionally, when an RhB aqueous solution without any added photocatalyst was irradiated with LED black light for the same duration, no change in absorbance was observed. This indicates that any decomposition of RhB would occur solely in the presence of a photocatalyst.

### 3.3. Degradation Experiments of MCN or UCN-Blended PVC Films

The method for film preparation is detailed in Section 3.3. In this experiment, we utilized an Everbeam LED black light, British Columbia, Canada (model US-365NM50W, 50 W). The wavelength distribution was measured with an Ocean Optics spectrometer MAYA2000PRO, Tokyo, Japan. A distinct peak was observed at 375 nm, with the emission extending from 365 to 400 nm. The light source was positioned 30 mm above the Petri dish, and the light intensity was consistently set to 6.0 mW cm^−2^ across all experiments (see Figure 5b).

Degradation was assessed through several methods: (i) visual observation of morphological changes, (ii) measurement of the weight loss rate of the film, (iii) surface analysis using a scanning electron microscope (SEM) from Hitachi High-Technologies, model SU-8000, (iv) quantification of chloride ions utilizing a Thermo Fisher Scientific Inc. Dionex Inuvion IC system, and (v) functional group analysis performed with a JASCO Co. FT/IR-4600 spectrophotometer. Additionally, changes in mechanical properties resulting from the incorporation of MCN or UCN were evaluated using an A&D Tensilon universal testing machine (RTG-1210).

### 3.4. Decomposition Experiment of MCN or UCN Mixed Electric Cable

As a representative example of e-waste, we selected a vinyl-insulated, vinyl-sheathed flat-type (VVF) cable (Ohm Electric, 1.6 mm × 2-core), which is commonly used in residential wiring. This cable consists of two copper wires enclosed in PVC insulation, all within a PVC sheath (see Figure 5c). In this study, we utilized the PVC sheath as the model coating material and constructed a pseudo-electric cable featuring a single copper wire insulated in PVC (the fabrication method is detailed in Section 2.5). For comparative purposes, we also created a pseudo-electric cable without UCN mixed in, which served as our control.

A Sunpie LED black light, California, United States (100 W) served as the primary light source for our experiments. Measurements of the wavelength distribution using an Ocean Optics spectrometer MAYA2000PRO, Tokyo, Japan indicated a sharp peak at 400 nm, with minimal emission of ultraviolet light. In addition to utilizing the LED light source, we also explored the decomposition behavior under sunlight. This outdoor experiment took place on the rooftop of Building No. 4 at Sophia University in Tokyo, regardless of weather conditions, from 25 October to 23 December 2024 (latitude 35.68426438, longitude 139.73153207). We positioned Petri dishes containing UCN cables and ion-exchanged water under eaves to protect them from rainwater.

## 4. Conclusions

This study introduced a self-degrading PVC wire sheath employing urea-derived g-C_3_N_4_ (UCN), a visible-light-responsive photocatalyst, for the recovery of copper from electronic waste. The UCN-PVC material demonstrated exceptional photocatalytic activity and effective decomposition while maintaining its strength and insulation in accordance with JIS standards. These findings indicate that self-degrading PVC coatings utilizing UCN may provide a viable solution to the challenges identified in our previous research, namely *enhancing visible light responsiveness* and *the presence of residual photocatalysts in the environment* [9]. Moreover, since power cables are typically installed in dark locations, such as within walls, the likelihood of light exposure is minimal. Even if light does reach the material, it will not decompose without the presence of water, and the decomposition process itself takes time, even under sunlight, thereby providing an effective safety measure. Future advancements, such as reducing the recombination rate of photogenerated electron-hole pairs in g-C_3_N_4_ [34] and enhancing the dispersibility of the photocatalyst, are anticipated to result in the creation of more efficient and practical self-degrading electrical wire coatings.

Interestingly, in parallel to our current study, Gou et al. [16] utilized graphitic carbon nitride (g-C_3_N_4_) as a photocatalyst to photodegrade polyvinyl chloride (PVC) under simulated sunlight, to assess its visible light photocatalytic capabilities. Their findings indicated that the organic nature and abundant surface functional groups of g-C_3_N_4_ facilitated its good dispersion in plastics. Notably, the ultrathin porous g-C_3_N_4_ nanosheet synthesized from urea (UCN) exhibited a significantly stronger photodegradation effect in PVC/g-C_3_N_4_ composite films compared to its melamine-synthesized counterpart (MCN). This aligns with our results, which demonstrate that the urea-derived, visible-light (l > 375 nm) responsive g-C_3_N_4_ is particularly effective at degrading PVC. Gou et al. [16] attributed this enhanced performance to the presence of superoxide radical anions (O_2_^−•^) and h^+^ generated by the UCN photocatalyst, which played a critical role in the photocatalytic dechlorination of PVC and the breakdown of its long-chain molecules into shorter, smaller ones. In contrast, we propose that, while direct decomposition of PVC by h^+^ may occur, it is likely less significant than the degradation resulting from the generated •OH radicals as shown earlier [9].

## Figures and Tables

**Table 1:** Variations in elemental composition resulting from the changes in abundance ratios of C (carbon), N (nitrogen), Cl (chlorine), and O (oxygen) on the surface of PVC film containing MCN (MCN/PVC film) or PVC film containing UCN (UCN/PVC film) before and after exposure to light irradiation.

	Mass Concentration/%			
	Surface of MCN/PVC Film		Surface of UCN/PVC Film	
Element	Initial Film	After 40 Days	Initial Film	After 40 Days
C	43.8	43.2	43.2	46.5
N	29.2	29.8	28.9	26.7
Cl	26.9	23.4	27.9	23.5
O		3.6		3.3

**Table 2:** Maximum stress, modulus of elastic modulus and elongation in tensile tests of UCN-containing PVC film and PVC film (without UCN).

Measurement Sample	Maximum Stress /MPa	Elastic Modulus/MPa	Elongation/%GL
UCN-containing PVC film	40.3	1142.8	6.4
PVC film (UCN-free)	38.4	100.3	12.0

**Table 3:** Maximum stress, modulus of elastic modulus and elongation in tensile tests of UCN-containing PVC sheath film and PVC sheath film (without UCN).

Measurement Sample	Maximum Stress /MPa	Elastic Modulus/MPa	Elongation/%GL
UCN (1 wt.%)/PVC film	1.1	0.4	595.6
UCN (5 wt.%)/PVC film	1.1	0.4	356.2
PVC film (UCN-free)	1.0	0.3	627.0

## Data Availability

Data are contained within the article.

## References

[1] Balde C.P., Kuehr R., Yamamoto T., McDonald R., D’Angelo E., Althaf S., Bel G., Deubzer O., Fernandez-Cubillo E., Forti V. The Global E-waste Monitor 2024. https://ewastemonitor.info/the-global-e-waste-monitor-2024/.

[2] https://www.env.go.jp/recycle/recycling/raremetals/conf_ruca/01/mat06.pdf.

[3] Basel Convention UNEP. https://www.basel.int/tabid/1275/Default.aspx.

[4] The Japanese Electric Wire & Cable Makers’ Association. https://www.jcma2.jp/toukei/index.html.

[5] S&P Global (2022). The Future of Copper: Will the Looming Supply Gap Short-Circuit the Energy Transition?. https://cdn.ihsmarkit.com/www/pdf/0722/The-Future-of-Copper_Full-Report_14July2022.pdf.

[6] Çetin A., Erzengin S., Alp F.B. (2019). Various combinations of flame retardants for poly(vinyl chloride). Open Chem..

[7] Wędrychowicz M., Kurowiak J., Skrzekut T., Noga P. (2023). Recycling of electrical cables—Current challenges and future prospects. Materials.

[8] Horikoshi S., Hachisuga N., Serpone N. (2024). Recycling of e-waste power cables using microwave-induced pyrolysis—Process characteristics and facile recovery of copper metal. RSC Adv..

[9] Horikoshi S., Serpone N., Hisamatsu Y., Hidaka H. (1998). Photocatalyzed degradation of polymers in aqueous semiconductor suspensions. 3. Photooxidation of a solid polymer:  TiO_2_-blended poly(vinyl chloride) film. Environ. Sci. Technol..

[10] United Nations Environment Assembly of the United Nations Environment Programme. https://www.env.go.jp/content/000045136.pdf.

[11] Dong S., Feng J., Fan M., Pi L., Hu L., Han X., Liu M., Sun J., Sun J. (2015). Recent developments in heterogeneous photocatalytic water treatment using visible light-responsive photocatalysts: A review. RSC Adv..

[12] Tang S.-B., Zhang S.-Y., Jiang Y.-X., Wang Z.-X., Li K., Luo Y.-Y., Long B., Su J. (2025). Visible-light-induced photocatalytic degradation of polyvinyl chloride under normal temperature and pressure via uranyl photocatalyst. Ind. Eng. Chem. Res..

[13] Kynman A.E., Christodoulou S., Ouellette E.T., Peterson A., Kelly S.N., Maron L., Arnold P. (2023). Photocatalytic dechlorination of unactivated chlorocarbons including PVC using organolanthanide complexes. Chem. Commun..

[14] Ran J., Talebian-Kiakalaieh A., Zhang S., Hashem E.M., Guo M., Qiao S.-Z. (2024). Recent advancement on photocatalytic plastic upcycling. Chem. Sci..

[15] Wang X., Maeda K., Thomas A., Takanabe K., Xin G., Carlsson J.M., Domen K., Antonietti M. (2009). A metal-free polymeric photocatalyst for hydrogen production from water under visible light. Nat. Mater..

[16] Gou N., Yang W., Gao S., Li Q. (2023). Incorporation of ultrathin porous metal-free graphite carbon nitride nanosheets in polyvinyl chloride for efficient photodegradation. J. Hazar. Mater..

[17] Kumar S., Karthikeyan S., Lee A.F. (2018). g-C_3_N_4_-based nanomaterials for visible light-driven photocatalysis. J. Hazard. Catal..

[18] Gupta A., Lakshmp Y.N., Manivannan R., Victoria S.N. (2017). Visible range photocatalysts for solid phase photocatalytic degradation of polyethylene and polyvinyl chloride. J. Chil. Chem. Soc..

[19] Cho S., Choi W. (2001). Solid-phase photocatalytic degradation of PVC–TiO_2_ polymer composites. J. Photochem. Photobiol. A Chem..

[20] Yang C., Gong C., Peng T., Deng K., Zan L. (2010). High photocatalytic degradation activity of the polyvinyl chloride (PVC)-vitamin C (VC)-TiO_2_ nano-composite film. J. Hazard. Mater..

[21] Fa W., Gong C., Tian L., Peng T., Zan L. (2011). Enhancement of photocatalytic degradation of poly(vinyl chloride) with perchlorinated iron (II) phthalocyanine modified nano-TiO_2_. J. Appl. Polym. Sci..

[22] Yang C., Deng K., Peng T., Zan L. (2011). Enhanced Solid-Phase Photocatalytic Degradation Activity of a Poly(vinyl chloride)-TiO_2_ Nanocomposite Film with Bismuth Oxyiodide. Chem. Eng. Technol..

[23] Yang C., Tian L., Ye L., Peng T. (2010). Enhancement of photocatalytic degradation activity of poly(vinyl chloride)-TiO_2_ nanocomposite film with polyoxometalate. J. Appl. Polym. Sci..

[24] Zhang Y., Sun T., Zhang D., Shi Z., Shi Z., Zhang X., Li C., Wang L., Song J., Lin Q. (2020). Enhanced photodegradability of PVC plastics film by codoping nano-graphite and TiO_2_. Polym. Degrad. Stab..

[25] Kaur K., Kaur H. (2025). Visible light induced photocatalytic degradation of polyvinyl chloride from water using graphene oxide/nitrogen-doped TiO_2_ nanocomposite. J. Environ. Chem. Eng..

[26] She X., Wu J., Zhong J., Xu H., Yang Y., Vajta R., Lou J., Liu Y., Du D., Li H. (2016). Oxygenated monolayer carbon nitride for excellent photocatalytic hydrogen evolution and external quantum efficiency. Nano Energy.

[27] Zhang M., Yang Y., An X., Zhao J., Bao Y., Hou L.A. (2022). Exfoliation method matters: The microstructure-dependent photoactivity of g-C_3_N_4_ nanosheets for water purification. J. Hazard. Mater..

[28] Sharma P., Sarngan P.P., Lakshmanan A., Sarkar D. (2022). One-step synthesis of highly reactive g-C_3_N_4_. J. Mater. Sci. Mater. Electron..

[29] Guo Q.X., Xie Y., Wang X.J., Lv S.C., Hou T., Liu X.M. (2003). Characterization of well-crystallized graphitic carbon nitride nanocrystallites via a benzene-thermal route at low temperatures. Chem. Phys. Lett..

[30] AEROXIDE® TIO2 P 25. https://www.coatino.com/products/PR_52043891.

[31] Hu Z.-Y., Liu T., Yang Y.-R., An A.K., Liew K.M., Li W.-W. (2025). Binder—Free immobilization of photocatalyst on membrane surface for efficient photocatalytic—H_2_O_2_ production and water decontamination. Nano-Micro Lett..

[32] Akerdi A.G., Mohsenzadeh M., Mahmoudian K., Bahrami S.H. (2025). A review on heterogeneous g—C_3_N_4_ for efficient treatment of contaminants: Fabrication, morphology control and environmental application. Inter. J. Environ. Sci. Techn..

[33] The Japanese Electric Wire & Cable Makers’ Association (JCMA). https://kikakurui.com/c3/C3605-2002-01.html.

[34] Wang Y., Shen S. (2020). Progress and prospects of non-metal doped graphitic carbon nitride for improved photocatalytic performances. Acta Phys. Chim. Sin..

